# Toddlers’ Active Gaze Behavior Supports Self‐Supervised Object Learning

**DOI:** 10.1111/desc.70231

**Published:** 2026-06-16

**Authors:** Zhengyang Yu, Arthur Aubret, Marcel C. Raabe, Jane Yang, Chen Yu, Jochen Triesch

**Affiliations:** ^1^ Frankfurt Institute for Advanced Studies Frankfurt Hessen Germany; ^2^ Xidian‐FIAS International Joint Research Center Frankfurt Hessen Germany; ^3^ Department of Psychology Center for Perceptual Systems University of Texas at Austin Austin Texas USA; ^4^ Department of Psychology University of California San Diego California USA

**Keywords:** computer vision, eye tracking, gaze behavior, object recognition, self‐ supervised learning, slowness learning

## Abstract

Toddlers learn to recognize objects from different viewpoints with almost no supervision. During this learning, they execute frequent eye and head movements that shape their visual experience. It is presently unclear if and how these behaviors contribute to toddlers’ emerging object recognition abilities. To answer this question, we here combine head‐mounted eye tracking during dyadic play with unsupervised machine learning. We approximate toddlers’ central visual field experience by cropping image regions from a head‐mounted camera centered on the current gaze location estimated via eye tracking. This visual stream feeds a neural network model, which uses a biologically plausible unsupervised learning objective. Our experiments demonstrate that a few minutes of such first‐person experience suffice to learn strong object representations permitting invariant object recognition. Importantly, by simulating alternative gaze behaviors we show that toddlers’ eye movement patterns play a crucial role in this. Our analysis also reveals that the limited size of the central visual field where visual acuity is high plays an important role for successful learning. Together, this highlights the benefits of temporally structured visual experience arising from toddlers’ natural interactions with objects.

**Summary:**

- We combine recordings of toddlers’ first‐person central visual field experience with biologically inspired self‐supervised learning algorithms to model toddlers’ development of invariant object recognition.
- Just a few minutes of toddlers’ central visual field experience captured with head‐mounted eye tracking suffice to learn strong object representations.
- Simulated alternative gaze behaviors produce weaker representations, demonstrating the importance of toddlers’ active gaze strategies for learning.
- Our results emphasize the importance of toddlers’ eye movements for learning object representations.

## 1 Introduction

Within their first year of life, toddlers learn to robustly recognize objects despite variations in viewpoints, lighting, and so forth (Kraebel and Gerhardstein 2006; Ayzenberg and Behrmann 2024). This early emergence of invariant object recognition and the ease with which adults perform this skill hide the complexities of acquiring it. For example, retinal images vary drastically when objects are rotated in depth and even state‐of‐the‐art machine learning methods make surprising recognition mistakes when faced with unusual viewpoints of objects (Dong et al. 2022; Abbas and Deny 2023; Ruan et al. 2023).

One of the main theories for how toddlers acquire invariant object recognition posits that their brains exploit a mechanism to construct visual representations that slowly change over time (Földiák 1991; Li and DiCarlo 2008; Miyashita 1988).

The computational principle of slowness learning, particularly as implemented in Slow Feature Analysis (SFA), is based on the assumption that although primary sensory inputs such as retinal pixel intensities change rapidly, the underlying semantic properties of the environment, such as object identity, vary much more slowly. By optimizing a slowness objective, the learning system is encouraged to discard information related to rapidly varying factors such as local illumination or object pose, while retaining temporally invariant features that remain stable over time. This temporal stability provides an unsupervised heuristic that enables the brain to associate different views of the same object into a coherent and viewpoint invariant representation (Franzius et al. 2011; Wiskott and Sejnowski 2002).

In the context of early childhood development, this computational framework aligns with the maturation of toddlers’ visual, motor, and cognitive systems. Developing toddlers actively structure their sensory environment through eye movements, manual object manipulation, and locomotion. These self‐generated physical interactions shape the temporal structure of visual experiences, and may provide the temporal stability of semantic information required for a slowness‐based learning mechanism to function.

Toddlers abundantly manipulate (or move around) objects while watching them, which gives them access to diverse views of a single object over a short period of time. By learning slowly changing representations, a toddler may be able to associate these different views, allowing them to form viewpoint‐invariant representations of objects (Wiskott and Sejnowski 2002; Schneider et al. 2021; Aubret et al. 2022a).

When processing visual input, humans typically select only a limited portion of the central visual field of a few degrees of visual angle for detailed analysis (Quaia and Krauzlis 2024; Yu et al. 2015; Zhaoping 2024). One reason for this is that receptor densities in the retina decline sharply towards the periphery (Jonas et al. 1992; Provis et al. 1985). As humans make on average around three saccades per second, the contents of the central visual field may be semantically unstable, that is, the central visual field may contain frequent transitions between different objects. This might interfere with a learning mechanism based on slowness. However, toddlers’ gaze behavior during interaction may stabilize the semantic content in their central visual field and thereby support learning via a slowness objective. For example, a learning toddler may not move their gaze randomly within a scene, but watch an object they are manipulating for an extended period of time before saccading to a different object. Previous models of visual representation learning in infants and toddlers have neglected the importance of eye gaze for learning (Orhan et al. 2020; Orhan and Lake 2024; Orhan et al. 2024). An exception to this is the work of Bambach et al. 2018, who, however, used a biologically implausible *supervised* learning approach.

Here, we investigate whether toddlers’ gaze behaviors may support the *unsupervised* learning of view‐invariant object representations. To this end, we leverage a dataset of head‐camera video recordings and eye gaze tracking from toddlers and adults during play sessions (Bambach et al. 2018). To simulate participants’ central visual field experience, we extract image patches centered on tracked gaze locations. This data feeds a computational model of a toddler's visual representation learning, which constructs representations that slowly change over time (Schneider et al. 2021; Aubret et al. 2022a). Our results show that toddlers’ gaze strategies boost visual learning in comparison to several baselines. Furthermore, we demonstrate that restricting learning to input from the central visual field improves the emerging object representations. Finally, we show that the visual input from toddlers permits learning better representations than that from adults. This finding is explained by toddlers looking longer at individual objects while manipulating them. Overall, by leveraging head‐mounted eye tracking and a neural network architecture with a biologically motivated unsupervised learning mechanism, our work represents the most advanced and precise model to date of the development of children's invariant object recognition abilities. It reveals how gaze behaviors and the principle of temporal slowness may jointly underpin the development of object recognition in children.

## 2 Methods

**FIGURE 1:**
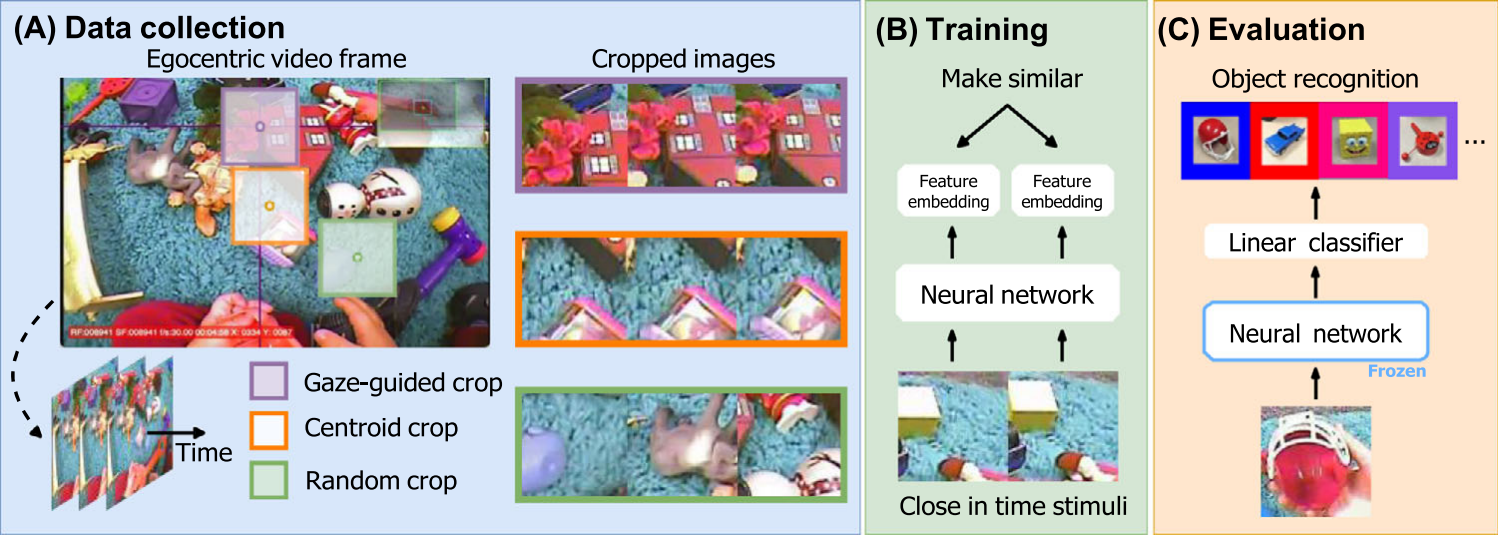
Overview of the experimental framework. (A) Eye‐tracking data from 38 toddlers and their caregivers were used. The resulting eye movement sequences formed a temporal dataset of visual observations. From each video frame, multiple crops are extracted using three methods to simulate the actual versus alternative central visual field experiences of participants: gaze‐guided crop (purple), centroid crop (orange), and random crop (green). Appendix A.1 provides a more detailed description of these three cropping methods. (B) We trained a computational model of biological visual learning to make the representations of temporally adjacent frames more similar. (C) To assess the quality of the learned visual representations, we train a linear classifier on top of the frozen trained neural network to perform object recognition.

Figure 1 provides an overview of the dataset, our computational model of toddlers visual learning, and the evaluation procedure used in this study. In the following sections, we briefly describe each component. More details are supplied in Appendix A.

### 2.1 Datasets

**FIGURE A1:**
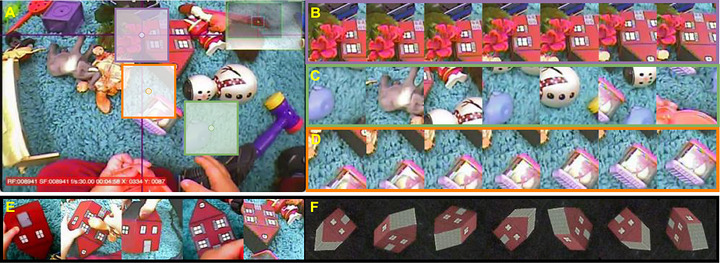
Examples of visual sequences for each of our datasets. (A) Raw frame from an egocentric video with the locations of our different croppings. Purple, orange, and green boxes represent the real‐time location of human eye movements, no eye movements, and random eye movements, respectively. The cross indicates the gaze location given by the eye‐tracker. (B–F) Example sequences for (B) the Toddlers’ Eye movements dataset; (C) the Random Eye movements dataset; (D) the No Eye movements dataset; (E) the Objects Fixation dataset and (F) the Blank Background dataset. Note that datasets (E–F) have been manually curated to only contain views of the target objects. This kind of oracle knowledge is not available to a naive learner.

We build on a dataset containing head‐camera videos and eye‐tracking data recorded from 38 dyads of toddlers and caregivers. Each video shows a dyad that plays with the same 24 toys for 15 min on average. The toddler participants had a mean age of 18.32 months (SD = 3.06 months, range = 12.3–24.3 months). To simulate the central visual field experience of a participant based on their eye gaze, we extract image patches (corresponding to 14°×14° of visual angle) from video frames of the scene camera mounted on the participant's head, which are centered on the recorded gaze position. Figure A1A shows an example of a sequence of image patches extracted around subsequent gaze locations (Bambach et al. 2018). We compare learning based on the visual input streams produced by these measured gaze behaviors (“Toddlers’/Adults’ eye movements”) of dyads against learning based on two simulated alternative gaze strategies. The first assumes that the camera‐wearer samples gaze locations uniformly at random within the field of view of the scene camera (“Random eye movements”). The second ignores eye movements (as in previous works) and instead assumes that the gaze location always remains in the center of the participant's field of view (“No eye movements”). In addition, we consider two idealized “oracles” that simulate a (biologically implausible) learner who fixates only on the objects and does not learn during transitions between objects. That is, this hypothetical learner already has perfect knowledge of when it is looking at an object of interest. We consider this strategy with natural backgrounds (“Object fixation”) and with blank backgrounds (“Blank background”). We show examples of visual sequences resulting from these strategies in Figure A1.

### 2.2 Computational Model

To model the learning process of humans, we train deep neural networks with two bio‐inspired self‐supervised learning models, namely SimCLR‐TT and BYOL‐TT (Schneider et al. 2021). Both models learn visual representations that associate close‐in‐time visual inputs. They also include a hyper‐parameter ∆*T* (measured in seconds) that quantifies how slowly representations should change. We use a ResNet18 as our default neural net‐ work architecture and provide additional results with a ResNet50 in Appendix B.4. Networks are trained “from scratch,” that is, they are not already pretrained for object recognition or any other task. Figure A1B illustrates the learning process. For each gaze dataset, a separate model was trained while keeping the neural network architecture, optimization procedure, and hyperparameter settings identical across conditions. Thus, differences in performance reflect differences in the visual input streams rather than differences in model architecture or training settings.

### 2.3 Evaluation

We train the models with video frames recorded from 30 randomly chosen dyads and keep the other 8 for testing. Even though recording durations varied substantially across toddlers, during training we pooled data across all toddlers’ videos rather than applying explicit normalization, subsampling, or weighting procedures. This choice was motivated by the goal of leveraging the full amount of available visual experience for model training.

We also consider training on the recording of single participants. We assess the quality of the learned representations by training a linear classifier on top of the learned representation (right after the average pooling layer) in a supervised fashion (Chen et al. 2020). Since our model of human visual representation learning does not use labeled images, we always train the linear classifier on the train split of the Objects Fixation dataset, which was manually labeled, and evaluate the object recognition accuracy on the test split of the Objects Fixation dataset. Statistical analyses were performed to assess the results. Unless otherwise specified, all significance tests were performed using two‐tailed independent two‐sample t‐tests. Correlation analyses were conducted using Pearson's correlation coefficient.

## 3 Results

### 3.1 Toddlers’ Central Visual Field Experience Supports the Learning of Invariant Object Representations via Time‐Based Self‐Supervised Learning

**FIGURE 2:**
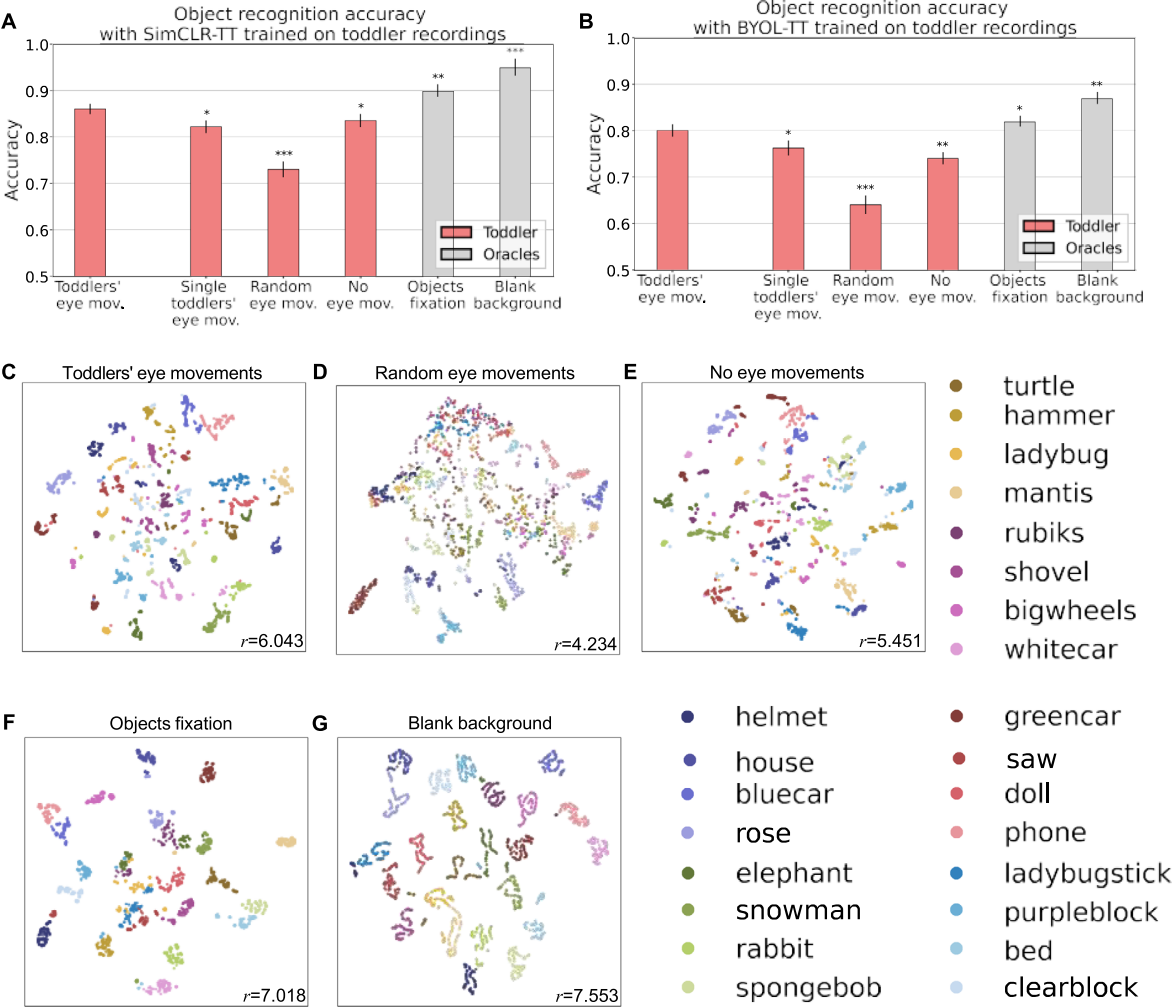
Toddlers’ gaze behavior supports the learning of invariant object representations. (A) and (B) show object recognition results of the linear classifier trained on top of representations learned with SimCLR‐TT and BYOL‐TT, respectively. Error bars represent the standard deviation over three random seeds. When training on data from just one toddler (“Single toddlers’ eye mov.”), we show the average accuracy and the standard deviation over the 38 toddlers. Results of statistical tests com‐ paring toddlers’ eye movements to other conditions are indicated as follows: “***”: *p*‐value *<* 0.001, “**”: *p*‐value *<* 0.01, and “*”: *p*‐value *< *0.05. (C–G) *t*‐SNE visualizations of learned object representations for SimCLR‐TT trained on different datasets. Each point represents an image sample, color‐coded by object identity (24 in total). The number in the bottom‐right corner of each panel displays the corresponding inter/intra‐cluster distance ratio computed in the full latent space at the end of training (higher is better, see Appendix B.2).

To test whether toddlers’ gaze behavior supports the learning of strong object representations, we compare the representations learned by the two unsupervised learning models based on slowness (see Appendix A.2 for details of SimCLR‐TT and BYOL‐ TT) when trained on the different datasets introduced in Section 2.1. Figure 2A and B show that models trained with the Toddlers’ Eye movements dataset outperform those trained with the Random Eye movements dataset or the No Eye movements dataset (*t*‐tests, *p < *0.05 in all cases). This suggests that toddlers’ gaze behavior supports the learning of view‐invariant object representations. A similar result is observed for adults (Appendix B.3).

Next, we study the impact of toddlers’ gaze behaviors on the learnt visual representations at a qualitative level. For each model trained using different eye movement strategies, we extract their representations of images in the Objects Fixation dataset and project these representations into a 2‐dimensional embedding space using t‐ SNE (Maaten and Hinton 2008). This allows us to visualize the (dis)similarity between representations of views of different objects (Figure 2C–G). The model trained on the Toddlers’ Eye movements dataset shows well‐separated clusters, indicating strong and discriminative object representations. In contrast, models trained with the Random or No Eye movements datasets produce less distinct clusters, reflecting weaker object learning. In contrast, the two biologically implausible “oracle” methods that train with the Objects Fixation dataset (F) or the Blank Background dataset (G) exhibit improved clustering.

Furthermore, we quantitatively assess the representation quality in the full latent space across conditions using inter/intra‐cluster distance ratios (higher is better; see bottom‐right of Figure 2C–G). Appendix B.2 explains how the inter/intra‐cluster distance ratio is calculated and how it evolves throughout training. Notably, the toddlers’ eye movements condition achieves the highest ratios among all eye movement strategies. This indicates that toddlers’ visual experience supports compact, well‐ separated representations of the different objects, whereas random eye movements yield lower ratios and a less clustered representational structure. The highest ratios are observed under the idealized but unnatural blank background and object fixation conditions.

We wondered whether the visual experience of only **a single** toddler during a play session suffices to build good visual representations. To investigate this question, we train SimCLR‐TT on the individual recordings of each toddler and compute the average of linear accuracies. We train the neural network using all fixation data from a single toddler, followed by training and testing the linear classifier with the Objects fixation data from the same and different toddlers. We control the training set to comprise 75% of the total data, ensuring that the test set does not overlap with the training set. Figure 2A and B show that the central visual experience of one toddler leads to representations almost as good as those resulting from the central visual experience of all toddlers.

### 3.2 Constraining Input to the Central Visual Field Improves Learning

Previous computational studies of learning from infants’ first person visual experience have neglected the importance of the constrained size of the central visual field for learning visual representations (Orhan et al. 2020; Sheybani et al. 2024). Here, we assess whether our simulated central visual field experience leads to better/worse object representations than learning from a wide field of view. We vary the size of the image portion extracted to simulate central vision. In Table 1, we observe for both toddlers and adults that an image size of 128 × 128 (corresponding to 14°× 14 of visual angle) produces the best recognition accuracy for all gaze strategies. Importantly, results for toddlers’ and adults’ eye movements at an image size of 128 × 128 pixels present an accuracy boost of 8% compared to the no eye movements condition for an image size of 480×480 pixels, which simulates head‐camera recordings without eye‐tracking information. We conclude that accounting for the constrained size of the central visual field is crucial for learning powerful object representations. We speculate that this boost results from the property of a 128 × 128 gaze‐centered crop to frequently capture the complete structure of an object while minimizing irrelevant background information, at least in the present dataset. However, the optimal crop size likely depends on additional factors such as object size, viewing distance, scene depth, and the amount of surrounding background entering the crop. These factors were not explicitly controlled in the present study and may influence how much object structure versus contextual information is preserved within the gaze‐centered image patches.

**TABLE 1:** Linear object recognition accuracy for different cropping sizes.

		64 *×* 64	128 *×* 128	240 *×* 240	480 *×* 480
Humans eye mov.	Toddler	0.831 ± 0.015	**0.863** ± **0.011**	0.828 ± 0.014	0.805 ± 0.018
	Adult	0.826 ± 0.013	**0.851** ± **0.028**	0.816 ± 0.013	0.791 ± 0.019
Random eye mov.	Toddler	0.701 ± 0.011	**0.736** ± **0.017**	0.694 ± 0.025	0.589 ± 0.036
	Adult	0.716 ± 0.021	**0.742** ± **0.022**	0.685 ± 0.023	0.576 ± 0.019
No eye mov.	Toddler	0.822 ± 0.016	**0.838** ± **0.010**	0.815 ± 0.018	0 *.* 784 ± 0 *.* 022
	Adult	0.818 ± 0.012	**0.829** ± **0.009**	0.807 ± 0.014	0 *.* 763 ± 0 *.* 017

*Note*: Best results in each row are highlighted in bold. Underlined results represent simulations that do not utilize actual gaze fixations and consider only the egocentric visual experience.

### 3.3 Toddlers’ Gaze Behavior Favors Stronger Emphasis on Slowness

**FIGURE 3:**
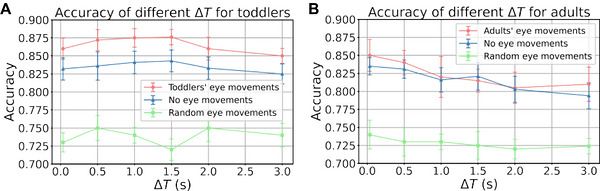
The impact of different ∆*T* on recognition accuracy for (A) toddlers and (B) adults. Error bars represent the standard deviation over three random seeds.

Semantic aspects of the visual experience have been shown to vary more slowly for toddlers than for adults (Sheybani et al. 2023). Our learning model includes a hyper‐parameter ∆*T* (measured in seconds) that quantifies how slowly representations should change. Concretely, it specifies the (maximum) time interval between two positive input pairs (whose representations will be made more similar) in the learning algorithms (see Appendix A.2 for the definition and implementation of ∆*T*). Interestingly, previous work has shown that increasing ∆*T* can improve the quality of object representations if visual inputs are sufficiently stable over time (Schneider et al. 2021; Aubret et al. 2022a). Thus, we wondered how changing ∆*T* may affect results and whether it may amplify any differences in the quality of representations learned from toddlers’ versus adults’ first person visual experience To test this, we varied ∆*T* from 1/30 to 3.0 s (Figure 3A). The models trained with the Toddlers' Eye movements dataset (red) achieve the highest recognition accuracy for an inter‐ mediate value of ∆*T *= 1.5s. In contrast, Figure 3B shows that, for models trained with the Adults’ Eye movements dataset, increasing ∆*T* only decreases the quality of the learned object representations. The results are consistent for both human eye movements (red) and the no eye movements datasets (blue). These results may reflect differences in gaze between toddlers and adults. Adults’ long gaze shifts and short fixation durations are more likely to bring their gaze to a different object within a few seconds. But making the representations of these views of different objects more similar is likely to make the resulting representation less good at object recognition, whereas toddlers’ longer object‐centered inspection periods may provide more tempo‐ rally stable object views that better support slowness‐based learning across larger ∆*T* (Franchak et al. 2016). We conclude that toddlers’ gaze behavior favors a stronger emphasis on slowness (greater ∆*T*) than that of adults.

### 3.4 Toddlers’ Long Object Inspections Relative to Adults Facilitate Learning

So far, we have shown that the gaze behaviors of humans support object learning via an unsupervised slowness objective and that toddlers gaze behavior favors a stronger emphasis on slowness (greater ∆*T*). We wondered what differences between toddlers’ and adults’ eye movements may contribute to this discrepancy. We analyzed four metrics that characterize the temporal sequence of images: the average fixation duration, the average duration of bouts of looking at the same object when not holding the object, the average duration of looking at an object when holding it, and the cumulative duration of object looking in an entire recording session. For these analyses, we leverage manually labeled timestamps (by Bambach et al. 2018) about when toddlers and adults look at/hold an object. See Appendix A.3 for details on saccade detection and calculation of fixation durations.

We successfully extracted the data from 28 out of 38 toddlers and conducted all subsequent experiments using these 28 toddlers. The remaining participants are excluded from this analysis due to the lack of data on fixation durations. Table D3 in Appendix D presents the details of the 28 included toddlers.

**FIGURE 4:**
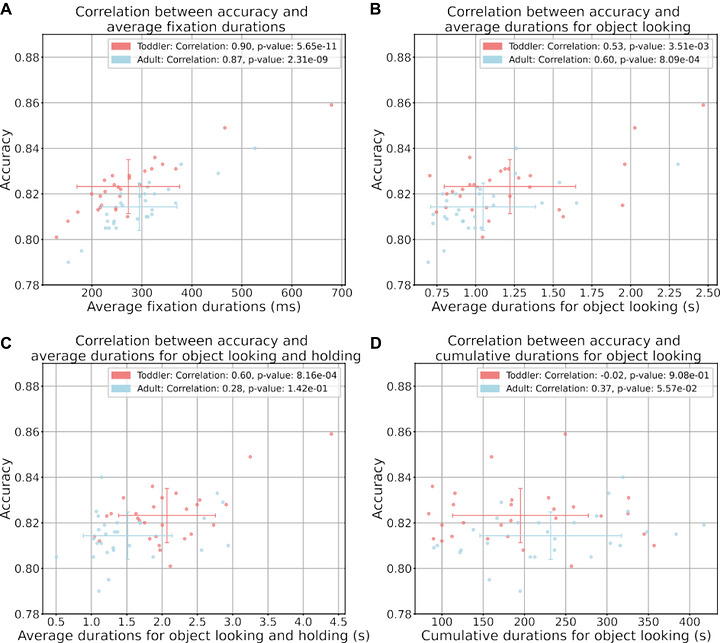
Correlation analysis between the recognition accuracy and (A) the average fixation duration; (B) the average duration of object looking while not holding the object; (C) the average duration of object looking while holding the object and (D) the cumulative duration of object looking. Models were all trained on the individual Toddlers’ and Adults’ Eye movements dataset. In each figure, the crosshairs represent the mean and standard deviation of the data values over the two axes. The legends show the Pearson correlation coefficients and their p‐values.

In Figure 4, we observe that object recognition accuracy is highly correlated with fixation durations, durations of object looking and duration of object looking while holding the object, but only weakly correlated with the cumulative duration of object looking. This indicates that long fixation bouts on the same object are important in explaining the relative quality of visual representations trained on Toddlers’ and Adults’ Eye movements datasets.

**FIGURE 5:**
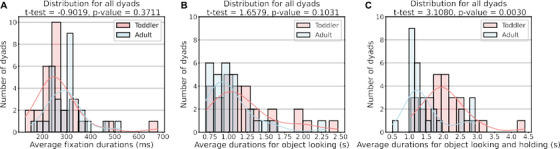
Comparison of (A) average fixation duration, (B) average duration of object looking while not holding the object, and (C) average duration of object looking while holding the object for toddlers and adults. Each panel includes the frequency distribution for the given metric, along with a density curve.

Figure 4 also shows that on average toddlers’ visual experience permits learning better representations than that of adults (*t*‐tests, *p *= 0.0053) with the given subset of dyads. To investigate which metric plays a crucial role in these differences. Figure 5 compares the distributions of average fixation durations for toddlers and adults. The t‐test statistics and p‐values are given in the titles. We observe that toddlers look longer at the object that they are holding (*t*‐test, *p* = 0.003). Other metrics do not exhibit statistically significant differences between adults and toddlers. We conclude that, compared to adults, toddlers’ longer periods of object inspection while manipulating objects allow for learning better viewpoint‐invariant object representations. More broadly, these findings raise questions about how embodied interaction structures temporal continuity in visual experience and how such structure may interact with slowness‐based learning objectives (see Appendix C for additional discussion).

## 4 Discussion

The mechanisms permitting infants and toddlers to acquire sophisticated object recognition abilities from very limited first person visual experience are still poorly understood. Here, we investigated whether neuro‐biologically motivated models of visual learning can take advantage of toddlers’ gaze behavior to develop robust object representations. We extracted toddlers’ gaze locations from egocentric video recordings with head‐mounted eye‐tracking during play sessions. By cropping image regions around these gaze locations we reconstructed the moment‐to‐moment central visual field experience of the toddlers. We used this data to train self‐supervised deep learning models with a biologically inspired slowness objective.

Our findings indicate that toddlers’ gaze strategies permit the learning of representations that support view‐invariant object instance recognition within a single play session of 12 min. Interestingly, models trained on adults’ visual experience performed significantly worse. Our analysis showed that focusing learning on inputs from the central visual field is beneficial for learning viewpoint‐invariant object representations and that toddlers’ gaze behavior favors a stronger emphasis on slowness compared to that of adults. This is consistent with toddlers looking longer at objects while holding them. During these relatively long holding periods, toddlers often turn and move the object, giving them access to sequences of different views of an object over a short period of time.

By leveraging head‐mounted eye tracking and a biologically motivated learning mechanism, our model captures the development of children's invariant object recognition abilities more precisely than previous works. For example, a study by Orhan & Lake used video data from a head‐mounted camera from a single child, but the data used for learning were sparse (only one video frame per minute) and without any eye tracking information (Orhan et al. 2020). An earlier study by Bambach et al. used head‐mounted eye tracking, but the model relied on a biologically implausible supervised learning approach (Bambach et al. 2018). Our work is unique and goes beyond such earlier approaches in that it attempts to accurately model infants’ learning from the moment to moment first per‐ son central visual field experience to the biologically inspired self‐supervised learning objective.

From a developmental perspective, our work provides strong evidence that the development of viewpoint‐invariant representations can originate from a slowness learning objective, a mechanism supported by neuroscientific studies (Li and DiCarlo 2008; Miyashita 1988). Our results also suggest that toddlers’ gaze behavior during naturalistic interaction generates temporally structured visual experience that supports learning of visual representations.

Furthermore, engaging with these computational principles allows us to address established developmental accounts of early object recognition. Developmental literature suggests that younger toddlers, often under 18 months, rely heavily on part‐ and feature‐based representations (Rakison 2003; Pereira and Smith 2009), whereas older toddlers, between 18 and 24 months, increasingly exhibit holistic, viewpoint‐invariant object representations (Smith 2003). We hypothesize that this representational transition may be functionally driven by systematic changes in infants’ own embodied behaviors over development. In early infancy, visual experience is relatively passive and fragmented. However, as toddlers mature, they develop more sophisticated object manipulation skills, such as holding, turning, and dynamically rotating objects closer to their eyes. This developmental milestone in active, coordinated hand‐eye behavior permits learning how, for example, controlled rotations of an object change its visual appearance. Under this view, the transition from feature‐based to holistic recognition observed in developmental psychology may not just be a matter of intrinsic neural maturation, but a direct consequence of the changing statistics of the sensory inputs curated by toddlers’ developing motor repertoires. In this work, we computationally test this hypothesis by demonstrating how such self‐generated, temporally continuous streams enable a self‐ supervised learning system with a slowness objective to bridge local, isolated features and bootstrap robust, holistic representations of object identity.

From a machine learning perspective, we show that combining head‐mounted eye‐ tracking video data with time‐based self‐supervised learning supports the emergence of viewpoint‐invariant object recognition. Our work therefore marks a step toward learning strong visual representations without handcrafted image datasets (e.g., Aubret et al. 2022a).

Our work also has several limitations. Because the adult recordings were obtained from caregivers engaged in dyadic interaction with toddlers, adults’ gaze behavior in our dataset may differ from adult visual exploration in more independently structured or non‐social object‐interaction settings. Thus, the present adult‐toddler comparisons should be interpreted within the specific context of caregiver‐child interaction.

Moreover, refining our approach to utilize both central and peripheral vision for learning visual representations could provide a more accurate simulation of human visual physiology and of the development of object versus scene representations in different brain areas. Our current cropping procedure represents a simplified approximation of foveated vision, since it completely removes peripheral information rather than preserving it at reduced spatial resolution. Peripheral vision nevertheless carries coarse but potentially informative visual signals (Quaia and Krauzlis 2024; Yu et al. 2015; Zhaoping 2024). Future work could therefore explore more biologically realistic approximations of foveated vision, such as graded peripheral blurring or continuous foveated representations, similar to approaches explored in previous computational studies of biologically inspired vision and gaze behavior (Wang et al. 2021).

We analyzed gaze behavior of toddlers with a minimum age of 12.3 months, meaning they had substantial visual learning experience before the experiment. In contrast, our computational models learned from scratch. Expanding to younger toddlers and a more diverse visual diet and distinct visual exploration patterns, could offer further insights into early visual representation development. Studying how babies under one year engage with objects may reveal new aspects of gaze behavior that contribute to their visual learning (Sheybani et al. 2024; Maurer 2017). Such attempts should also be guided by knowledge of infants’ developing contrast sensitivity. Regarding the learning mechanism, while the slowness objective used in our model is biologically plausible, the detailed implementation via backpropagation of errors in a deep neural network is not. It is an interesting challenge to replace this mechanism with a more biologically realistic alternative. Finally, a more complete model of visual learning in infants and toddlers needs to also capture the computational mechanisms driving their gaze shifts, whose relevance for visual representation learning we have demonstrated here. Understanding these mechanisms will be an important next step in unraveling the mechanisms underlying the early development of human visual perception.

Finally, We Note that our focus on slowness learning was motivated by the present data and research question: the learning needs to extract relatively stable object representations from continuously changing viewpoints during naturalistic interaction. Toddlers’ visual experience during object manipulation often contains temporally stable object‐centered views over multi‐second intervals, making slowness‐based learning particularly well matched to the temporal statistics of the dataset.

At the same time, slowness learning is related to alternative temporal learning frameworks such as predictive coding and prediction‐based learning (Földiák 1991; Rao and Ballard 1999). However, the two approaches emphasize somewhat different objectives. Predictive coding and other prediction‐based learning approaches aim to generate accurate predictions of future sensory input, whereas slowness learning emphasizes suppressing rapidly changing fluctuations in appearance in order to extract stable latent structure (Földiák 1991; Rao and Ballard 1999; Lotter et al. 2016). These mechanisms may therefore play complementary roles in biological vision systems and may contribute differently across ventral and dorsal processing streams.

The ventral stream, associated with object identity and recognition, may benefit more from a slowness objective that abstracts away transient appearance changes. The dorsal stream, associated with spatial processing and motion analysis, may instead rely more heavily on precise prediction of visual dynamics (Goodale and Milner 1992). Future work incorporating both objectives within a unified model could provide a more complete account of the development of visual representation across these distinct cortical pathways.

## Author Contributions

**Arthur Aubret**: conceptualization, investigation, writing – original draft, methodology, visualization, writing – review and editing, software, supervision, formal analysis. **Zhengyang Yu**: conceptualization, investigation, writing – original draft, writing – review and editing, visualization, methodology, software, formal analysis, validation. **Marcel C. Raabe**: conceptualization, investigation, writing – original draft, methodology, visualization, writing – review and editing, software, formal analysis, validation. **Jochen Triesch**: funding acquisition, writing – original draft, supervision, conceptualization, writing – review and editing, project administration. **Chen Yu**: conceptualization, funding acquisition, writing – original draft, writing – review and editing, project administration, supervision. **Jane Yang**: data curation, methodology, writing – review and editing.

## Ethics Statement

All procedures involving the collection of data from toddlers and their caregivers were conducted at the Developing Intelligence Lab in the Department of Psychology at the University of Texas at Austin, and the collection was reviewed and approved by the Institutional Review Board at their institution.

## Conflicts of Interest

The authors declare no conflicts of interest.

## Permission to Reproduce Material From Other Sources

No copyrighted material from other sources was used in this manuscript.

## Figures and Tables

**FIGURE A2:**
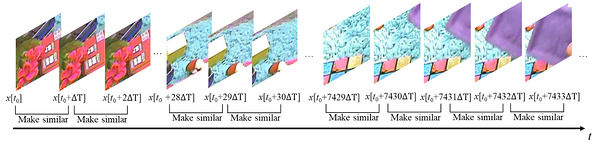
Supp: Illustration of SimCLR‐TT on the Toddlers’ Eye movements dataset. By default, the time interval ∆*T* = 1/30 s corresponds to the inverse of the camera's frame rate, but it can be increased to an integer multiple of this value.

**FIGURE B3:**
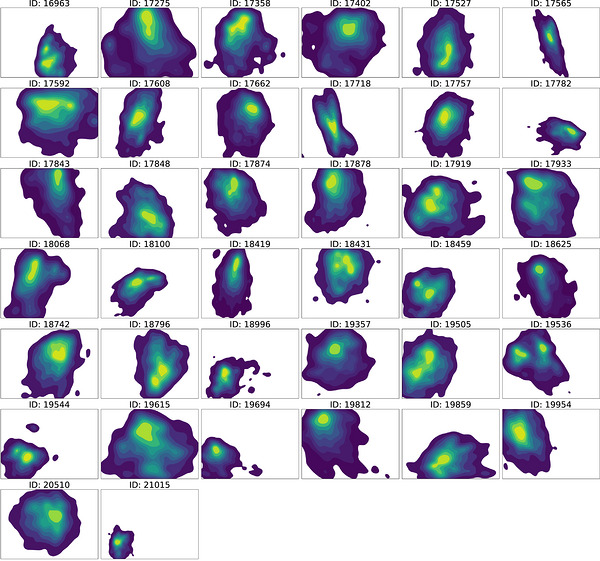
Gaze distribution of individual toddlers during dyadic play. Each subplot shows a toddler's 2D gaze distribution (kernel density estimate) across a play session. Warmer colors indicate higher gaze density.

**FIGURE B4:**
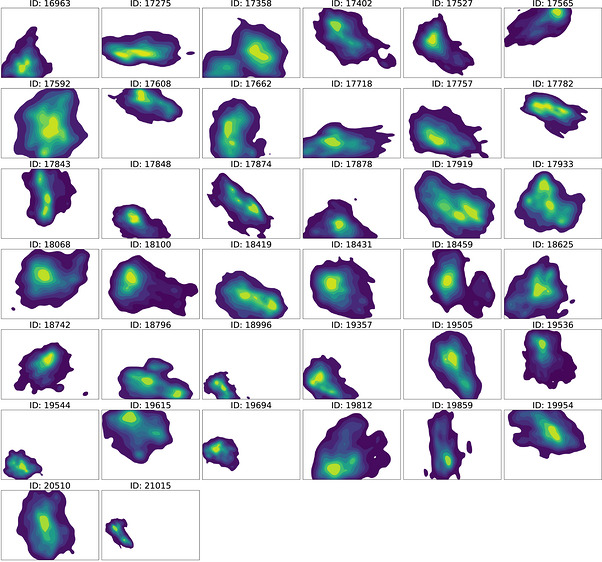
Gaze distribution of individual adults during visual exploration. Each subplot shows an adult's 2D gaze distribution (kernel density estimate) across a play session. Warmer colors indicate higher gaze density.

**FIGURE B5:**
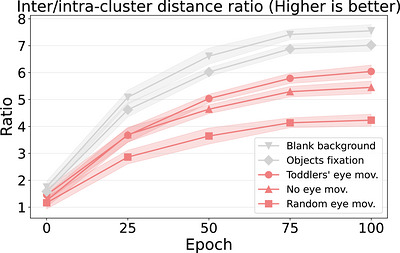
Inter/intra‐cluster distance ratio as a function of number of training epochs for five conditions. All feature representations were L2‐normalized before distance computation. Lines represent the mean, and shaded areas denote the standard deviation across three independent runs. Data points were recorded at 25‐epoch intervals.

**FIGURE B6:**
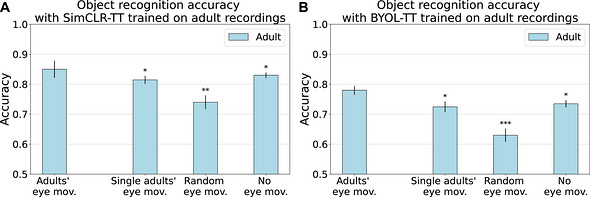
Linear object recognition accuracy of training on a single adult and all adults. (A) and (B) show the results for SimCLR‐TT and BYOL‐TT applied to the adult recordings, respectively. The error bars represent the standard deviation over three random seeds. When training on one adult, we show the average accuracy and the standard deviation over the 38 adults. We also provide the significance test results for adults’ eye movements and other groups. “***” indicates *p*‐value *<* 0.001, “**” indicates *p*‐value *< *0.01, and “*” indicates *p*‐value *< *0.05.

**FIGURE B7:**
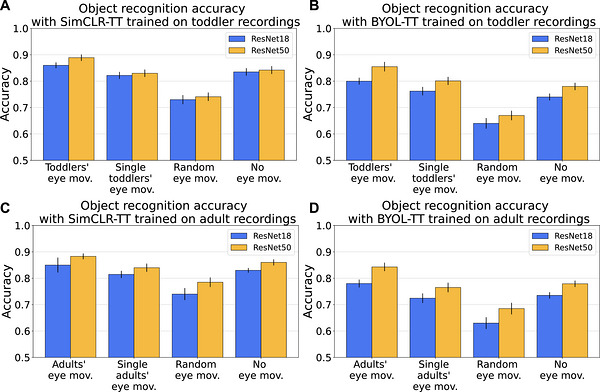
Different encoder training on individual toddler/adult and all toddler‐ s/adults. (A) and (B) show the results for SimCLR‐TT and BYOL‐TT applied to toddlers’ datasets, respectively; (C) and (D) present the corresponding results when switched to adults’ datasets. Error bars represent one standard deviation over three random seeds.

**FIGURE B8:**
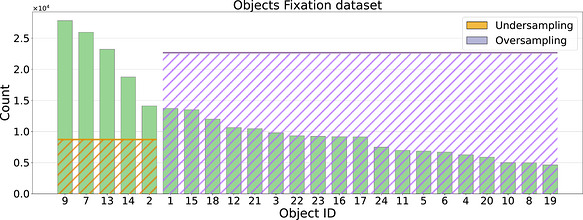
Sampling on the objects fixation dataset. Refer to the Tables D1 and D2 for the full object names corresponding to each ID. Among the top 5 most frequent objects, the yellow regions indicate their sample counts after undersampling. These, combined with all samples from the remaining objects, form the Undersampling dataset. The purple regions indicate the oversampled counts for the less frequent objects, which, together with all original samples from the top 5 objects, constitute the Oversampling dataset. The upper bounds of the yellow and purple regions correspond to the average sample counts for the non‐top‐5 and top‐5 objects, respectively. The resulting dataset sizes are 204,462 (undersampling), 271,754 (original), and 527,449 (oversampling).

**FIGURE B9:**
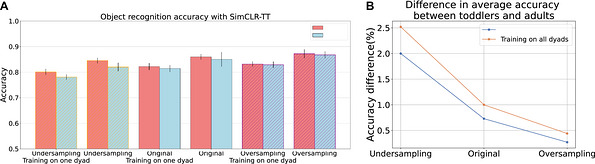
Linear object recognition accuracy and the difference in accuracy between undersampling and oversampling. (A) We compared the recognition accuracy under different sampling methods. Additionally, we provide the test results after training on one dyad versus all dyads. Error bars represent the standard deviation over three random seeds; (B) The difference in recognition accuracy between toddlers and adults under different sampling methods. Here, we also compare the accuracy differences of the model trained on one dyad versus all dyads.

**FIGURE B10:**
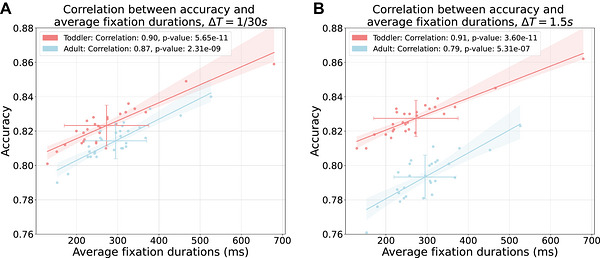
∆*T* highlights the differences between toddlers and adults. We observe variations in the test accuracy of models trained on the Toddlers’ and Adults’ Eye movements dataset under (A) ∆*T =* 1/30s and (B) ∆*T* = 1.5s. We attached fitted regression lines, and the shaded areas show the 95% confidence interval. The crosshairs represent the mean and standard deviation of the data values over the two axes.

**FIGURE B11:**
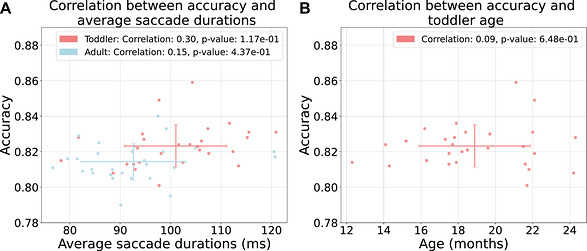
The impact of (A) average saccade duration and (B) toddler's age on recognition accuracy. The crosshairs represent the mean and standard deviation of the data values over the two axes.

**FIGURE B12:**
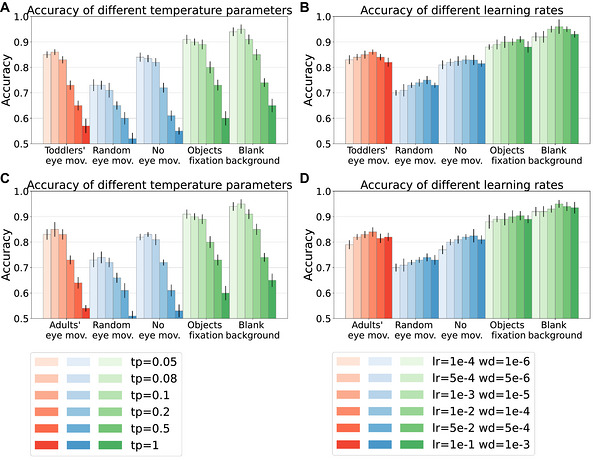
Object recognition accuracy across different hyper‐parameter settings. (A) and (B) show the results for different learning rates and temperature parameters applied to toddlers’ datasets, respectively, (C) and (D) present the corresponding results when switched to adults’ datasets. The vertical bars represent the standard deviation over three random seeds.

## Data Availability

The data supporting our analyses were obtained from the private dataset described in (Kraebel and Gerhardstein 2006). Anyone interested in accessing the data may contact the Developing Intelligence Lab at The University of Texas at Austin (chenyulab@austin.utexas.edu) for further assistance. The Python code used for data processing (including different cropping strategies), analysis, and results in this study is publicly available on GitHub (https://github.com/trieschlab/infantVision).

## Appendix A Method Details

### A.1 Datasets

The used dataset (Bambach et al. 2018) contains head‐camera videos recorded at 30 frames per second (FPS) and eye‐tracking data for 38 dyads of toddlers and caregivers. All dyads play with the same set of 24 toys for 12 min on average. The toddlers’ ages range from 12.3 to 24.3 months. For 30 dyads, a head‐camera resolution of 640×480 pixels was used, while four dyads were recorded at 720×480 pixels and the remaining four at 320×240 pixels. The horizontal field of view covers 72 degrees.

Although recordings differed somewhat in pixel resolution, all cameras shared approximately the same horizontal field of view (72°). Thus, the gaze‐centered crops were designed to approximate a consistent visual angle (14°×14°) across participants rather than an identical physical pixel density. As a consequence, some variability in effective image detail remained across recordings, especially for the smaller subset of lower‐resolution videos, which represents a limitation of the dataset.

Figure A1A shows an example video frame with the gaze location (Bambach et al. 2018). In the following, we explain how we simulate different gaze strategies by deriving several datasets from these videos and gaze positions. Additionally, we include the anonymized information of all toddlers who participated in the study in Appendix D.

**Toddlers’ Eye movements dataset**. This dataset aims to simulate the central visual experience of toddlers. We cut out an image patch centered on the current gaze point. For the cut‐out's size, we choose 128×128 pixels as the default, which corresponds to 14°×14° of visual angle. A typical temporal sequence of this dataset is illustrated in Figure A1B. If the gaze fixation point is too close to the image border, the crop boundaries may extend beyond the image, making it impossible to extract a patch of the desired size. In this case, we shift the gaze fixation point from the problematic border orthogonally by the minimum number of pixels. This ensures that the cropping operation outputs an image with the correct size. Note that the cropped area always contains the actual gaze fixation point. This dataset contains 559,522 training images. This number is consistent across all eye movement datasets (see below).

**Adults’ Eye movements dataset**. This dataset was derived from the care‐ givers participating in the same dyadic play sessions as the toddlers. We want to investigate the differences between gaze fixation in adults and toddlers and the con‐ sequences of these differences on learned representations. Thus, we also extract image patches around adults’ gaze fixation points following the procedure of the Toddlers’ Eye movements dataset. Appendix B illustrates the gaze distributions of toddlers and adults.

**Random Eye movements dataset**. As a comparison dataset, we simulate a completely random gaze strategy. We crop each frame around a location that is sampled uniformly at random. Unlike the Toddlers’/Adults’ Eye movements datasets, this dataset shows little spatio‐temporal correlation structure, and the cropped images are unlikely to contain well‐centered objects. Figure A1D provides example frames from the Random Eye movements dataset.

**No Eye Movements Dataset**. We Also Propose a Stronger Comparison Dataset That Considers a human Moving Their Head but Not Their Eyes. This Is an Important comparison because it distills the effect of eye movements. One possibility for building such a dataset could be to always crop the center of the frames. However, we noticed that the head‐camera was often misaligned with respect to the stationary position of the eyes, resulting in a mismatch between the center of the frames and the center of the camera wearer's field of view (cf. Appendix B). Thus, we rather use the centroid of the gaze fixation points (one for each participant video). To compute these centroids, we gather all gaze fixation points of a subject and calculate the mean of their horizontal and vertical coordinates. Note that, despite the centroid positions being fixed, the continuous movements of the head change the visible portions of the scene. Nevertheless, compared to the Random Eye movements dataset, this set contains image patch sequences that are relatively stable over time. Figure A1D presents a temporal sequence of the No Eye movements dataset.

We also consider two “oracle” datasets that were constructed using the ground truth about an object's identity/location. Models trained on this dataset aim to upper‐ bound our model's performance.

**Objects Fixation dataset**. This dataset was collected from the same video frames used in the Toddlers’ Eye movements dataset. Images were manually filtered so that toddlers looked at one of the target objects. From these frames, crops with a 30‐degree field of view around the gaze location were extracted, containing the tar‐ get object while minimizing background interference (Bambach et al. 2018; Tsutsui et al. 2021). This dataset contains 271,754 images. Figure A1E displays examples of images.

**Blank Background Dataset**. This Dataset Contains 1,536 Images, Capturing each Object from 64 Possible Viewpoints with Various Angles and Distances. All Toy Models in this dataset originate from the Object Fixation dataset. Each image displays a complete object against a black background, ensuring visual isolation from external distractions. Figure A1E shows an example toy from different viewpoints.

### A.2 Learning Models

To model the learning process of humans, we learn visual representations with self‐ supervised models that construct similar representations for close‐in‐time visual input.

**SimCLR‐TT**. This algorithm is based on the state‐of‐the‐art SimCLR method (Chen et al. 2020). SimCLR‐TT samples an image *x_t_* at time *t* and a temporally close image *x_t_*_+∆_ *_T_* and computes their respective embeddings *z_t_*, *z_t_*_+∆_ *_T_* with a deep neural network (Schneider et al. 2021) (e.g., a ResNet).

Our Model Pairs Images whose Temporal Distance Is Not More than the Parameter ∆*T*. Unless stated otherwise, we set ∆*T* to the inverse of the camera's frame rate, that is, ∆*T* = 1/30 s, corresponding to the smallest temporal interval directly observable in the recorded video stream. In the context of slowness learning, ∆*T* determines the temporal range over which visual inputs are encouraged to develop similar representations. Larger ∆*T* values therefore impose stronger temporal invariance constraints across longer timescales. The underlying assumption is that temporally adjacent visual inputs are more likely to correspond to the same object undergoing gradual viewpoint or appearance changes, such that enforcing representational continuity across time may support the emergence of viewpoint‐invariant object representations. In Section 3.3 we show additional results varying ∆*T*. Then, SimCLR‐TT minimizes

(A1)
$$L\left(z_{t},z_{t+\Delta T}\right)=-\log\frac{\exp(\text{sim}\left(z_{t},z_{t+\Delta T}\right)/\tau )}{\sum_{z_{k}\in B,k\neq t}\left[\exp\left(\text{sim}\left(z_{t},z_{k}\right)/\tau \right)\right]},$$

where ℬ is a minibatch, sim(·) is the cosine similarity and τ is the temperature hyper‐parameter. Here k≠t but k=t+δT is possible. Thus, SimCLR‐TT maximizes the similarity between temporally close representations (numerator) while keeping all representations dissimilar from each other (denominator). Figure A2 illustrates the learning process of SimCLR‐TT.

**BYOL‐TT**. To evaluate whether our conclusions also hold for different methods that learns with temporal slowness, we perform the same experiments with BYOL‐ TT. Similarly to SimCLR‐TT, BYOL‐TT was originally considered to be used for contrastive learning through time (Schneider et al. 2021). Its loss function is defined as

(A2)
$$L_{\theta_{t},\;\xi_{t+\Delta T}}=2-2\cdot (\mathrm{sim}\left(q_{\theta_{t}}\left(z_{\theta_{t}}\right),z_{\;\xi_{t+\Delta T}}\right),$$

where $q_{\theta_{t}}\left(z_{\theta_{t}}\right)$ is the prediction of the online network for one frame and $\;z_{\;\xi_{t+\Delta T}}$ represents outputs from the target network. The weights of the online network are denoted by *θ*, and *ξ* represents the weights of the target network. Again, we use the cosine similarity as the similarity function. Appendix B provides details on the training and evaluation procedures.

Importantly, Fixation and Saccade Were Not Explicitly Incorporated into the Self‐ supervised Learning Procedure. Instead, the Learning Models Operated Directly on the continuous temporal sequence of gaze‐centered visual inputs, and temporally adjacent image pairs were sampled according to the temporal interval parameter ∆*T*.

### A.3 Extraction of Saccades and Fixations

The analyses in Section 3.4 include extracting fixation bouts. This requires detecting saccades, as they mark possible beginnings and ends of the fixation bouts. To detect saccades in gaze movements, we apply a velocity threshold‐based method similar to (Raabe et al. 2023). Consecutive gaze points that exceed a threshold *T*_1_ are identified as a single saccade. To account for artifacts caused by low frame rates, a second threshold *T*_2_, along with an angular criterion *θ*, allows the inclusion of the two data points adjacent to the saccade initially detected. Any data points not classified as saccades are considered fixations. For this study, we choose *T*_1_ = 25°s*^−^* ^1^, *T*_2_ = 10 s*^−^* ^1^ and *θ* = 45 °.

### A.4 Neural Network Training Details

For each condition, we train multiple models with identical parameters but different randomly initialized weights. We then report average results with error bars indicating one standard deviation across the three random initializations (three random seeds). For each seed, we split the 38 available dyads into 30 train dyads and 8 test dyads. This quantifies how reliable the results are for one particular data set and algorithm with a specific set of hyperparameters. This variability is taken into account when statistically comparing results for different datasets or algorithms or hyperparameter values. This procedure allows us to estimate variability across random initializations and assess the robustness of the learned representations.

We train the models on train dyads for 100 epochs with a ResNet18, the AdamW optimizer and set the initial learning rate and weight decay to 10*^−^* ^2^ and 10*^−^* ^4^, respectively. We set the SimCLR temperature to 0.08 and the batch size to 256. Appendix B.8 presents the results under various settings of hyper‐parameters. We conduct all experiments on an Nvidia GeForce RTX 3090 GPU with 24 GB memory.

## Appendix B Complementary Analyses

### B.1 Gaze Location Distribution

In Section A.1, we explain that the center of the frames is misaligned with respect to the stationary position of the eyes. To support this statement, Figures B3 and B4 display the distribution of gaze locations for each toddler and adult, respectively. Brighter areas indicate higher frequencies of gaze fixation at those locations. The results indicate that the average gaze location is typically not centered with respect to the camera.

### B.2 Inter/Intra‐Cluster Distance Ratios During Training

To evaluate the quality of learned representations, we trained an encoder using the SimCLR‐TT framework. Every 25 epochs during training, the encoder was frozen and used to extract L2‐normalized features from the Objects fixation dataset. These features were used to compute the inter/intra‐cluster distance ratio, which quantifies how well the learned representation separates images of different objects.

Specifically, the intra‐cluster distance measures the average pairwise distance between the feature representations of images of the same object, while the inter‐ cluster distance measures the average distance between representations of images of different objects. A higher ratio therefore indicates more compact within‐object clustering and clearer separation between the representations of different objects, reflecting a stronger, more clustered representational structure.

More precisely, the **intra‐cluster distance** is defined as the average pairwise distance between all samples belonging to the same object:

(B3)
$$D_{\mathrm{intra}}=\frac{1}{\sum_{o}N_{o}\left(N_{o}-1\right)}\sum_{o}\sum_{\begin{array}{c} i,j\in o\\ i\neq j \end{array}}d(Z_{i},Z_{j}),$$

where $z_{i}\in R^{d}$ are L2‐normalized encoder features, *N_o_* is the number of samples for object *o*, and $d\left(\cdot ,\cdot \right)$ denotes Euclidean distance.

The **Inter‐cluster Distance** Is Computed across all Pairs of Samples from Different objects:

(B4)
$$D_{\mathrm{inter}}=\frac{1}{\sum_{o\neq o^{\prime }}N_{o}N_{o^{\prime }}}\;\sum_{o\neq o^{\prime }}\sum_{i\in o}\sum_{j\in o^{\prime }}d\left(z_{i},z_{j}\right),$$

where *o*′ denotes a different object from *o*. The final ratio is defined as:

(B5)
$$\text{Ratio}=\frac{D_{inter}}{D_{intra}}.$$

As shown in Figure B5, all five training conditions led to progressively increasing ratios over time. The toddlers’ eye movements consistently achieved the highest ratios among all eye movement‐based strategies.

### B.3 Adults’ Central Visual Field Experience Also Supports the Learning of Invariant Object Representations

To examine the suitability of adults’ looking behavior for learning viewpoint‐invariant object representations, we replaced the toddlers’ datasets with adults’ data and applied the same evaluation procedure as described in Section 3.1. The results in Figure B6 suggest that biologically inspired visual learning models like SimCLR‐TT and BYOL‐ TT can also leverage adult gaze behavior to learn invariant object representations. The central visual field experience of one adult leads, on average, to representations almost as good as that of the combined central visual field experiences of all adults. These findings are in line with the conclusion about toddlers in Section 3.1.

### B.4 Impact of Changing the Self‐Supervised Learning Encoder

We compared the accuracy of BYOL‐TT and SimCLR‐TT for ResNet18 versus ResNet50 encoders on both the Toddlers’ and Adults’ datasets. As shown in Figure B7, the more complex ResNet50 encoder resulted in an improved accuracy, especially for BYOL‐TT.

### B.5 Object‐Wise Sample Imbalance in the Objects Fixation Dataset

Toddlers and adults may spend different times looking at different objects. In Figure B8, we show the number of images per toy, sorted in descending order. We observe in the Objects Fixation dataset that some objects are more present than others.

To investigate the impact of this object sample imbalance, we adjusted the distribution of the Objects Fixation dataset while keeping the encoders pre‐trained in Section 3.1. The linear classifier was then trained and tested on the adjusted datasets. We compared two approaches for addressing sample imbalance across object categories:

**Undersampling**. We applied random undersampling to reduce the number of samples in the top 5 categories, making their quantities similar to those of the other categories.

**Oversampling**. Similarly, we applied random oversampling to increase the number of samples in the underrepresented categories to match the quantity of the top 5 categories. Compared to “Undersampling”, this method results in duplicate samples in the dataset.

In Figure B9, we observe that when the total sample size is reduced, the recognition accuracy of the models trained on Toddlers’ and Adults’ Eye movements dataset decreases, but the difference in their accuracy continues to widen. However, with more complex or balanced training, the model's generalization capacity improves, and the performance across toddlers and adults tends to converge, reducing the impact of differences in visual behaviors.

### B.6 Further Differences Between Toddlers and Adults

We provide additional evidence highlighting the differences between toddlers and adults. In Figure B10A and B, we compare the changes in recognition accuracy for both toddlers and adults under different ∆*T* values. From the regression lines, the increasing ∆*T* amplifies the difference in recognition accuracy between training on Toddlers’ and Adults’ Eye movements datasets, consistent with the findings in Section 3.3.

### B.7 Study of Saccade Duration and Age

Here, we complement Section 3.4 and study two additional metrics that may impact the performance of individual adults and toddlers, namely the average saccade duration and toddlers’ age. According to Figure B11, we observe no significant correlation between the recognition accuracy and either the average saccade duration or toddlers’ age. However, the youngest toddlers in the study were older than one year, and we cannot rule out that younger babies may show different results.

### B.8 Robustness Under Varying Hyper‐Parameters

The learning rate (lr), weight decay (wd), and temperature (tp) used in our main experiments were selected as the best settings after a hyper‐parameter search. To assess the robustness of our method, we conduct additional experiments where we set lr = 10*^−^* ^2^, wd = 10*^−^* ^4^, tp = 0.08, and vary hyper‐parameters individually. As shown in Figure B12, changes in these hyper‐parameters do not affect the conclusions presented in Section 3.1.

## Appendix C Embodied Interaction and Slowness Learning

### C.1

An important consideration is that temporal continuity may not simply reflect toddlers’ slowly varying visual input, but may instead be actively structured through embodied interaction (Yu and Smith 2012). Actions such as holding, manipulating, rotating, and tracking objects can maintain object visibility across extended temporal windows, reduce abrupt changes in retinal input, and stabilize objects within the toddler's field of view. In this sense, temporal continuity may arise partly from the toddler's own sensorimotor behavior.

This perspective is relevant for the interpretation of our findings, because temporal slowness alone does not uniquely specify object identity. Slowly varying features may also reflect stable background structure, viewing conditions, or other correlated regularities in natural scenes. Although the inter‐/intra‐cluster analyses suggest greater representational consistency and separation, these metrics do not by them‐ selves establish fully stable object identity representations. Rather, our results suggest that temporal continuity may provide one constraint that supports the emergence of more coherent representations across time.

Embodied interaction may additionally alter the nature of the learning signal itself. During manual exploration, changes in object appearance are often linked to self‐ generated actions, producing lawful and predictable transformations across viewpoints and moments in time. Such sensorimotor structure may provide stronger constraints for representation learning than passive temporal adjacency alone, potentially facilitating the development of viewpoint‐tolerant and temporally coherent representations (Xu and Triesch 2023; Aubret et al. 2024). More broadly, these considerations suggest that developmental learning may depend not only on the temporal statistics of sensory input, but also on how toddlers actively sample and organize experience through perception and action (Aubret et al. 2022b).

A related limitation of the present study is that our analyses focus primarily on visual temporal statistics and do not explicitly model the action‐perception loop through which toddlers generate much of their experience. Future work incorporating hand‐object interactions, gaze behavior, or manipulation dynamics may help clarify how embodied interaction contributes to the temporal slowness learning and the emergence of stable representations during early development.

## Appendix D Details of all Toys and Toddlers Data

### D.1

We provide information for all toys and toddlers participating in the study in Tables D1, D2, and D3. The toddler ID represents an anonymized identifier for each toddler.

**TABLE D1:** 24 toys were used for toddler interaction. See Table D2 for the second part. Among them, “Library” refers to those toys that were success‐ fully recognized when the toddler calls any word from the corresponding row. However, these columns are not within the scope of the current study's discussion. The main focus is on the colors, shapes, or textures of these 24 toys, which are more likely to help toddlers differentiate between them.

Gazetag naming	ICONS	ID	Library
helmet		1	helmet, hat
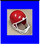
house		2	house, home
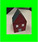
bluecar		3	car
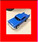
rose		4	rose, flower, plant
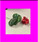
elephant		5	elephant
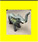
snowman		6	snowman
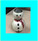
rabbit		7	rabbit, bunny
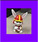
spongebob		8	spongebob, block
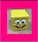
turtle		9	turtle, tortoise
hammer		10	hammer, tool, mallet
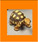
ladybug		11	bug, insect, ladybug, beetle
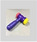
mantis		12	bug, insect, praying mantis, mantis, grasshopper
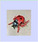

**TABLE D2:** 24 toys were used for toddler interaction. See Table D1 for the first part.

Gazetag naming	ICONS	ID	Library
greencar		13	car
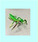
saw		14	saw, tool
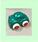
doll		15	baby, baby doll, girl, doll
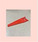
phone		16	phone, telephone
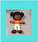
rubiks		17	block, rubiks cube, rubiks, cube
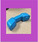
shovel		18	rake, shovel, tool
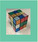
bigwheels		19	truck, jeep, bigwheel, car
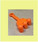
whitecar		20	car, policecar
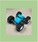
ladybugstick		21	ladybug, bug
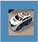
purpleblock		22	block, cube
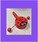
bed		23	Bed
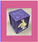
clearblock		24	block, cube

**TABLE D3:** Information for each participating toddler (Anonymized).

Toddler ID	Frame count	Video length (min.:sec.)	Age (months)	Gender	Resolution (px^2^)
16,963	16,440	9:07	20.7	M	720 *×* 480
17,275	9120	5:04	18.2	F	720 *×* 480
17,358	18,930	10:31	18.8	M	720 *×* 480
17,402	27,636	15:21	19.2	M	640 *×* 480
17,527^‐^	15,242	8:28	21.5	M	640 *×* 480
17,565^‐^	14,864	8:15	19.7	F	640 *×* 480
17,592^‐^	16,116	8:58	18.2	M	640 *×* 480
17,608^‐^	18,059	10:02	21.8	F	640 *×* 480
17,662^‐^	14,553	8:05	15.2	F	640 *×* 480
17,718	11,850	6:35	18.1	F	720 *×* 480
17,757^‐^	19,661	10:55	21.7	F	640 *×* 480
17,782^‐^	9035	5:01	22.1	F	640 *×* 480
17,843^‐^	18,209	10:07	19.6	F	640 *×* 480
17,848^‐^	21,111	11:43	18.4	F	640 *×* 480
17,874^‐^	17,429	9:41	17.8	M	640 *×* 480
17,878^‐^	20,018	11:08	17.5	F	640 *×* 480
17,919^‐^	18,596	10:20	22.1	M	640 *×* 480
17,933^‐^	14,457	8:02	17.9	F	640 *×* 480
18,068^‐^	7976	4:26	17.9	M	640 *×* 480
18,100^‐^	14,982	8:19	16.3	F	640 *×* 480
18,419^‐^	28,253	15:41	17.3	M	640 *×* 480
18,431^‐^	11,575	6:26	22	M	640 *×* 480
18,459^‐^	7231	4:01	16.2	F	640 *×* 480
18,625^‐^	18,209	10:07	24.3	F	640 *×* 480
18,742^‐^	19,018	10:34	17.7	M	640 *×* 480
18,796^‐^	11,672	6:30	24.2	M	640 *×* 480
18,996	12,466	6:56	15.9	F	320 *×* 240
19,357^‐^	8834	4:54	17.5	M	640 *×* 480
19,505^‐^	18,397	10:13	18.5	M	640 *×* 480
19,536^‐^	18,370	10:13	21.1	M	640 *×* 480
19,544	9151	5:05	13.8	F	320 *×* 240
19,615^‐^	13,351	7:25	14.1	M	640 *×* 480
19,694	10,801	6:00	15.2	M	320 *×* 240
19,812^‐^	9918	5:31	21.6	M	640 *×* 480
19,859	7360	4:05	14.4	M	640 *×* 480
19,954^‐^	9201	5:07	12.3	F	640 *×* 480
20,510^‐^	11,865	6:35	14.35	M	640 *×* 480
21,015	9566	5:19	13	M	320 *×* 240

*Note*: IDs marked with “ * ” indicate inclusion in Section 3.4. Frame Count denotes the total video frames used; Video Length refers to the recording duration. Age represents the participant's age at recording. Gender is marked as M (male) or F (female). Resolution indicates the video resolution from the head‐mounted camera.
